# Pattern of congenital heart diseases in Rwandan children with genetic defects

**DOI:** 10.11604/pamj.2014.19.85.3428

**Published:** 2014-09-25

**Authors:** Raissa Teteli, Annette Uwineza, Yvan Butera, Janvier Hitayezu, Seraphine Murorunkwere, Lamberte Umurerwa, Janvier Ndinkabandi, Anne-Cécile Hellin, Mauricette Jamar, Jean-Hubert Caberg, Narcisse Muganga, Joseph Mucumbitsi, Emmanuel Kamanzi Rusingiza, Leon Mutesa

**Affiliations:** 1 Department of Pediatrics, Kigali University Teaching Hospital, University of Rwanda, Kigali, Rwanda; 2 Center for Medical Genetics, School of Medicine and Health Sciences, University of Rwanda, Huye, Rwanda; 3 Center for Human Genetics, Centre Hospitalier Universitaire Sart-Tilman, University of Liège, Liège, Belgium; 4 Department of Clinical Genetics, Kigali University Teaching Hospital, University of Rwanda, Kigali, Rwanda; 5 Medical Student, College of Medicine and Health Sciences, University of Rwanda; 6 Department of Pediatric Cardiology, King Faysal Hospital, Kigali, Rwanda; 7 Department of Pediatric Cardiology, Kigali University Teaching Hospital, University of Rwanda, Kigali, Rwanda

**Keywords:** Congenital heart disease, genetic defects, pediatric patients, Rwanda

## Abstract

**Introduction:**

Congenital heart diseases (CHD) are commonly associated with genetic defects. Our study aimed at determining the occurrence and pattern of CHD association with genetic defects among pediatric patients in Rwanda.

**Methods:**

A total of 125 patients with clinical features suggestive of genetic defects were recruited. Echocardiography and standard karyotype studies were performed in all patients.

**Results:**

CHDs were detected in the majority of patients with genetic defects. The commonest isolated CHD was ventricular septal defect found in many cases of Down syndrome. In total, chromosomal abnormalities represented the majority of cases in our cohort and were associated with various types of CHDs.

**Conclusion:**

Our findings showed that CHDs are common in Rwandan pediatric patients with genetic defects. These results suggest that a routine echocardiography assessment combined with systematic genetic investigations including standard karyotype should be mandatory in patients presenting characteristic clinical features in whom CHD is suspected to be associated with genetic defect.

## Introduction

Congenital heart disease (CHD) is a problem of heart's structure and function that is present at birth. It can describe a number of different anomalies affecting the heart. It is the most common type of birth defect [1]. Population based studies on the prevalence of CHD worldwide was found to range between 1.0- 150 per 1000 livebirths [1]. CHD is often divided into two groups, one of cyanotic heart disease such as tetralogy of Fallot (TOF), truncus arteriosus (TA), transposition of the great arteries (TGA); and acynotic heart disease including especially ventricular septal defect (VSD), atrioventricular septal defect (AVSD), patent ductus arteriosus (PDA), atrial septal defect (ASD), pulmonary stenosis (PS), and coarctation of the aorta (CoA) [1, 2]. The CHDs are classified as multifactorial defects but the overwhelming majority of congenital heart malformations do not segregate in Mendelian ratios, although they show familial aggregation, which suggests that genetic factors may play a role in their development [3]. Most of the known causes of CHDs are sporadic genetic variations, point mutations, deletion or duplication [4]. Moreover, a large spectrum of chromosomal abnormalities such as Trisomies 21, 13, and 18 are associated with CHD in 5-8% of cases [5, 6]. In addition, a small proportion of chromosomal abnormalities are also frequently related to CHD, and the most common chromosomal abnormality found in this group is the 22q11 microdeletion causing DiGeorge syndrome [7]. Almost 30% of major cardiac malformations are associated with additional developmental abnormalities and result from a recognized chromosomal abnormality syndrome or occur as part of a genetic syndrome [8, 9]. However, up to now few data on CHD associated with genetic defects have been reported in African populations [7]. In addition, only one study done in Rwanda in 2007 showed some cases of Down syndrome associated with CHD and the VSD was the most common [10]. Apart from Down syndrome, many other genetic diseases such as Noonan syndrome are known to be associated with CHD [11]. Moreover, a recent clinical case study of Rwandan patients with Noonan syndrome found all patients to be affected with CHD and PS was the most frequent [12]. Previous research evidences have shown that the prevalence and pattern of group of genetic disorders associated with CHD vary both within and between regions and countries [13, 14]. For instance, we observe a high prevalence of this association in Africa. This is mainly due to the unfavourable socio-economic conditions in Africa, the lack of diagnostic genetic laboratories and also prenatal diagnosis to prevent genetic diseases. Therefore, the aim of the present study contributes to determine the occurrence and pattern of CHD associated with genetic abnormalities in pediatric patients using available cytogenetic diagnostic and echocardiographic facilities in Rwanda.

## Methods

### Patients

Our study population was composed of a group of 125 patients from birth to 15 years; recruited over two years from May 2010 and May 2012. All enrolled patients were selected based on the presence of dysmorphic features and/or other clinical signs suggesting of any genetic defect documented to be associated with CHD; also consulting the department of pediatrics at Kigali University Teaching Hospital (KUTH) during the study period (Table 1). Then patients meeting the above criteria were addressed to the department of genetic clinics for further investigations. This study was approved by the Institutional Review Board (IRB) of KUTH. The parents signed an informed consent before collecting blood samples from patients. They also provided a signed consent for publication of patients’ photos.

**Table 1:** Summary of clinical features of different syndromes

Clinical findings	Trisomy 21	Trisomy 18	Trisomy 13	Cat Eye	Turner	Williams	DiGeorge	Deletion 13 qter	Other Syndromes*	Total
(n= patients’ number)	n = 89	n = 7	n = 4	n = 2	n = 1	n = 1	n = 1	n = 1	n = 19	n = 125
*Clinical features*										
Psychomotor Delay	83	7	4	-	-	1	1	1	16	113
Hypotonia	72	4	4	-	-	1	-	1	1	83
Hyperflexibility	49	-	-	-	-	-	-	-	-	49
Microcephaly	39	2	3	-	-	-	-	1	5	50
Uncurled hair	73	5	3	1	-	-	-	-	2	84
Flat Facial profile	62	-	-	-	-	-	-	-	1	63
Hypertelorism	85	5	3	2	-	1	1	1	14	112
Mongoloid Slant	81	-	1	-	-	-	-	-	2	84
Epicanthal folds	81	-	1	-	-	-	1	1	2	86
Nystagmus	1	-	-	-	-	-	-	-	-	1
Strabismus	9	-	2	1	-	-	-	1	1	14
Microphtalmia	-	1	3	2	-	-	-	-	2	8
Coloboma	-	-	-	1	-	-	-	-	-	1
Ear abnormalities	78	7	5	-	-	-	1	-	13	104
Preauricular tags	-	-	-	2	-	-	-	-	-	2
Flat nasal bridge	71	2	2	2	-	-	1	1	5	84
Protuding tongue	46	-	-	-	-	-	-	-	1	47
Proeminent Upper Lip	-	-	-	-	-	-	-	-	5	5
Cleft lip/palate	-	-	2	-	-	-	-	-	-	2
Micro/retrognatia	8	7	1	-	-	-	-	-	5	21
Short neck	76	5	2	-	1	-	-	1	11	96
Excessive neck skin	55	-	2	-	1	-	-	-	7	65
Chest Deformity	11	-	-	-	-	-	-	-	4	15
Wide spaced nipples	27	2	1	-	1	-	-	-	6	37
Brachydactyly	49	-	-	-	-	-	-	-	-	49
Clinodactyly	-	1	-	-	-	-	-	-	1	2
Clenched hand	-	5	2	-	-	-	-	-	2	9
Syndactyly	-	-	-	-	-	-	-	-	3	3
Polydactyly	-	1	4	-	-	-	-	-	-	5
Simian crease	41	2	1	-	-	-	-	1	1	46
Wide sandle Gap	47	-	-	-	-	-	-	-	1	48
Rocker bottom foot	-	5	-	-	-	-	-	-	1	6
*Gastro-intestinal anomalies*										
Umbilical hernia	22	2	3	-	-	1	-	-	1	29
Anus atresia	1	-	-	1	-	-	-	-	1	3
Duodenal Atresia	1	-	-	-	-	-	-	-	-	1
Hirshprung disease	3	-	-	-	-	-	-	-	-	3
*Urogenital anomalies*										
Cryptorchidism	2	1	-	-	-	-	-	1	-	4
Hypospadias	-	-	-	-	-	-	-	1	-	1

* Represents other syndromes identified in the present study which include 4 cases of Noonan syndrome, 1 case of Poland syndrome, 1 case of Fraser syndrome, 1 case of Cornelia de Lange as well as 13 cases

### Echocardiography

The echocardiography was performed using a SIEMENS ACUSON X300 Machine. The 2D and M mode were used to determine the heart anatomy, the size of heart chambers and the function of both right and left ventricles. The study with color Doppler allowed assessing the level of regurgitation and velocity through atrioventricular valves, semilunar valves and the great vessels. Transducers with 9 and 4 MHz were selected and used according to the age and the size of the patients.

### Cytogenetic and molecular analysis

Standard karyotype was performed in all patients on Q-banded metaphases spreads prepared from peripheral blood cells following conventional protocols. We used 500 resolution's level for banding characterization. DNA samples were extracted and sent to the Center for Human Genetics in Liege-Belgium for further molecular testing. Multiplex Probe Ligation Amplification (MLPA) was performed in all patients with normal karyotype using the SALSA MLPA P245 microdeletion syndrome kit and SALSA MLPA P070/ P036 for human telomere screening (MRC-Holland, Amsterdam, The Netherlands). Fluorescence in Situ Hybridization analysis (FISH) was performed to confirm 22q11 deletion (Di George Syndrome), 7q11.23 (Williams’ syndrome). Lymphocytes were cultured by standard methods. Samples were analyzed by using dual-color FISH probes specific to the commonly deleted area. Signals were visualized using a fluorescent microscope. Between fifty metaphases and 200 interphase nuclei from patient were evaluated.

## Results

**Figure 1:**
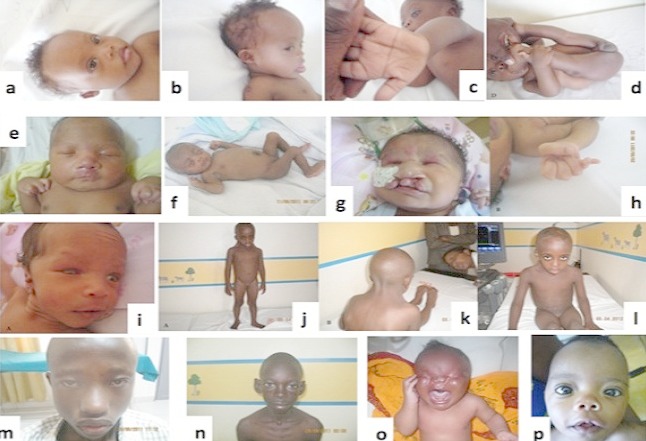
Patient photos with characterized different syndromes. Patients affected by Down syndrome. (a) hypertelorism, flat nasal bridge, short neck, mongoloid slant; (b) low set ears and protruding tongue; (c) single palmar crease and (d) hyperflexibility; (e) Patient affected by Edwards syndrome, note the presence of hypertelorism, flat nasal bridge, short neck, clenched hands with overlapping fingers; (f) umbilical hernia and club feet; (g) Patient affected by Patau syndrome with typical facial dysmorphism characterized by hypertelorism, cleft lip and palate; (h) and polydactyly; (i) Patient with Cat eye syndrome showing right preauricular tag, right microphthalmia and coloboma; (j) Patient affected by Turner syndrome presenting with short stature; (k) webbed neck and (l) wide spaced nipples; (m) Patient affected by DiGeorge syndrome. Note the presence of long face, hypertelorism, proeminent nose with squared nasal root; (n) Patient affected by Williams-Beuren syndrome presenting periorbital fullness (puffiness around the eyes), long philtrum, wide mouth, large and protruding ears; (o) Patient with Fraser syndrome showing cryptophthalmos, ears abnormalities and syndactyly; (p) Patient affected by Noonan syndrome with physical facial signs showing hypertelorism, long philtrum and proeminent upper lip

A systematic karyotype was performed in all 125 patients enrolled in this study and MPLA screening was done in all remaining patients with normal karyotype. The standard karyotype was abnormal in 103 patients (Table 2). The most frequent chromosome abnormality detected in this group was trisomy 21 found in 89 patients. Among these cases, 84 had free trisomy 21 due to meiotic non disjunction while 5 cases were due Roberstonian translocation in which we detected 3 cases of translocation between both chromosomes 21, one of t(21,22)(q10;q10) and another one of t(13,21)(q10,q10). In addition, we identified 4 cases of Trisomy 13 (Patau syndrome), 3 with free trisomy 13 whereas 1 was a Roberstonian translocation between both chromosomes 13; t(13,13)(q10,q10). We also found 7 cases of free trisomy 18 (Edwards syndrome). Two cases of Cat eye syndrome including a mosaic form and 1 case of Turner syndrome were also detected. Figure 1 shows different patients with clinical features characteristic of the identified syndromes.

**Table 2:** Cytogenetic and molecular findings in pediatric patients recruited in the present study

Diagnostic	Genetic results	N° of cases	Percentage (%)
	*Karyotype result*		
Free Trisomy 21	47,XX,+21 or 47, XY, +21	84	67,2
Trisomy 21 with Roberstonian translocation	46, XX der(21; 21)(q10;q10)	1	0,8
	46, XY der(21; 21)(q10;q10)	2	1,6
	46,XX, 21rob(21;22)(q10;q10)	1	0,8
	46,XY, 21rob(13;21)(q10;q10)	1	0,8
Free Trisomy 18	47,XX,+18 or 47, XY, +18	7	5,6
Free Trisomy 13	47,XX,+13 or 47, XY, +13	3	2,4
Tisomy 13 with Roberstonian translocation	46,XX, 13rob(13;13)(q10;q10)	1	0,8
Cat Eye syndrome	47, XX,+mar22	1	0,8
Cat Eye syndrome	47, XX,+mar22/46,XX	1	0,8
Turner Syndrome	45,X0	1	0,8
Total of abnormal karyotype		**103**	**82,4%**
	***MLPA result***		
Williams Syndrome	7q11.23 deletion	1	0,8
DiGeorge Syndrome	22q11.2 deletion	1	0,8
Deletion 13qter	46, XY,-13qter	1	0,8
Total of abnormal MLPA results		3	2,4%
Normal karyotype and MLPA result	46,XX or 46,XY	19	15,2
Total		125	100

**Figure 2:**
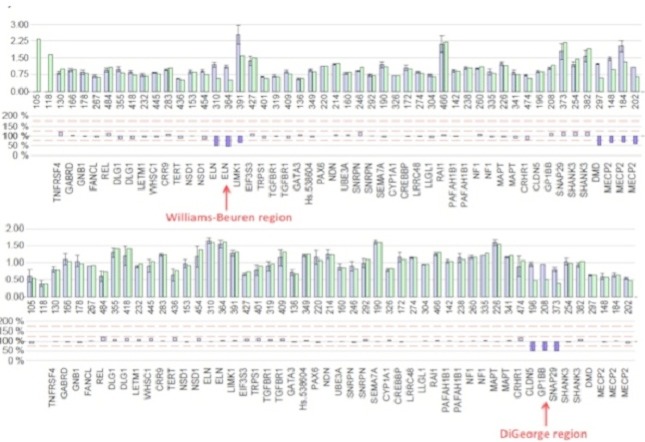
MLPA results. On the above chart, the red arrow shows deletion of two probes ELN and LIMK1 involved in Williams-Beuren syndrome (microdeletion 7q11.23), whereas the red arrow on the below figure shows the deletion of three probes, i.e. CLDN5, GP1BB and SNAP29 involved in DiGeorge syndrome

**Figure 3:**
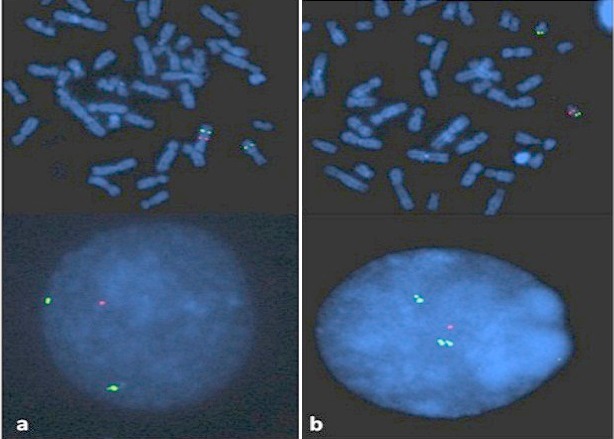
FISH analysis performed on interphase nuclei and metaphase mitoses showing: a) the absence of 1 red signal on one of the two 7q11.23 chromosomes in Williams-Beuren syndrome; b) the absence of 1 red signal which indicates the deletion of 22q11 in DiGeorge syndrome

MLPA analysis performed in 22 patients with normal karyotype revealed one case of deletion of 7q11.23 region involved in Williams-Beuren syndrome, one case of deletion 13qter and one case of patient presenting 22q11.2 microdeletion causing DiGeorge syndrome) (Figure 2). Fluorescence In Situ Hybridization (FISH) analysis was used to confirm those cases (Figure 3). In total, chromosomal abnormalities represented 84,8% in our cohort (Table 2). In addition, 19 patients had a normal karyotype and a normal MLPA results. However, in this group we detected clinically 4 patients with suggestive phenotype of Noonan syndrome, 1 case of Cornelia de Lange, 1 case of Fraser syndrome and 1 case of Poland syndrome.

The echocardiography was performed in 125 patients and 61 children had normal heart anatomy while CHD was diagnosed in 64 children. Among the diagnosed children with CHDs, 48 presented a single cardiac defect, while 16 had associated cardiac defects. Table 3 summarizes all types of identified CHD in different genetic syndromes and their frequencies. The commonest isolated CHD was patent ductus arteriosus (PDA) affecting 14 patients including 13 cases of Down syndrome. Other frequent isolated CHDs were ventricular septal defect (VSD) and artrioventricular septal defect (AVSD) also predominant in Down syndrome patients. There were two cases of dextrocardia identified in Poland syndrome and Down syndrome, respectively. Complexe cases of CHDs with association of atrial septal defect (ASD) and truncus arteriosus (TA), PDA with ventricular septal defect (VSD), and VSD combined to pulmonary valvular stenosis (PS) were detected in 16 patients whose the majority were affected by Down syndrome.

**Table 3:** Frequency of CHDs in different genetic syndromes

Echocardiac findings	Genetic Disorders													
	Trisomy 21(n = 89)	Trisomy 18(n = 7)	Trisomy 13(n = 4)	Cat Eye (n = 2)	Turner (n = 1)	Williams (n = 1)	Di George (n = 1)	Deletion 13qter (n = 1)	Noonan (n = 4)	Poland (n = 1)	Cornelia de Lange (n = 1)	Fraser (n = 1)	Normal karyotype & MLPA	Frequency of CHD type
***Isolated CHD (N = 48)***														
ASD	3	-	-	-	-	-	-	-	1	-	-	-	-	4
AVSD	4	-	-	-	-	-	-	-	1	-	-	-	-	5
CoA	-	-	-	-	1	-	-	-	-	-	-	-	-	1
Dextrocardia	1	-	-	-	-	-	-	-	-	1	-	-	-	2
MVP	-	-	-	-	-	1	-	-	-	-	-	-	-	1
PDA	13	-	1	-	-	-	-	-	-	-	-	-	-	14
PS	1	-	-	-	-	-	-	-	1	-	-	-	-	2
TOF	2	1	-	-	-	-	1	-	-	-	-	-	-	4
TA	1	-	1	-	-	-	-	-	-	-	-	-	-	2
VSD	9	3	1	-	-	-	-	-	-	-	-	-	-	13
***Sub Total***														48
***Associated CHD (N = 16)***														
ASD + TA	4	1	-	-	-	-	-	-	-	-	-	-	-	5
PDA + VSD	9	-	1	-	-	-	-	-	-	-	-	-	-	10
VSD + PS	1	-	-	-	-	-	-	-	-	-	-	-	-	1
**Sub Total**														16
***Absence of CHD (N = 61)***														
Patients with normal														
Echocardiography	41	2	0	2	0	0	0	1	1	0	1	1	12	61
Sub Total														**61**
**TOTAL**														**125**

n = number of patients with genetic defect; N =frequence number of CHD types; ASD = Atrial Septal Defect; AVSD = Atrioventricular Septal Defect; CoA = Coarctation of Aorta; MVP =Mitral Valve Prolapse; PDA = Patent Ductus Arteriosus; PS = Pulmonary Stenosis; TOF = Tetralogy of Fallot; TA = Truncus Arteriosus; VSD = Ventricular Septal Defect

## Discussion

The CHDs are mostly associated with genetic disorders and often caused by chromosomal abnormalities, point mutations, deletion or insertion in specific genes. Previous studies with large cohort it have been shown that chromosomal abnormalities and other genetic defect associated with extra-cardiac malformations occurred in nearly one fourth of CHD cases in the population [15–17]. A recent study done in Lagos found 68% of recognized genetic syndromes to be associated with CHDs [18]. In this study, authors assessed patients with clinical approach but nevertheless they did not perform any genetic investigations. The most common syndrome so far identified in this study was Down syndrome in 78.3% of the enrolled patients. Other clinically identified syndromes and associations were Marfan′s, Noonan′s, Edwards, Prune Belly, Apert, Ellis-van Creveld and congenital rubella syndromes. In our cohort, echocardiographic studies detected 64 patients with CHDs in whom genetic defects were found. Among the genetic defects observed in our study, the chromosomal abnormalities were detected in the majority of cases and Trisomy 21 was predominant representing 71,2% of all recruited cases. Interestingly, previous other studies performed in children with genetic defects associated with CHD showed a similar incidence of Down syndrome ranging between 40-60% [5, 6, 19]. In South African populations few studies have shown high incidence of CHD in patients with Down syndrome [20, 21]. Also, Ghana showed same findings of high rate of CHD in clinically diagnosed Down syndrome patients [22]. In our study, among the 89 patients with Down syndrome the echocardiography revealed CHDs in 53 patients. The VSD, AVSD and PDA were the most frequent isolated heart defects. These findings were in accordance with the above reported studies where they showed that congenital cardiac defects mainly AVSD, VSD, PDA, and ASD represent the most frequent clinical findings in Down syndrome [5, 10, 19, 23]. Among other chromosomal abnormalities identified in our patients, Edwards syndrome was also predominant. Based on clinical features, we identified 7 cases of Edwards syndrome among them 3 had VSD, which is the most common CHD occurring in 90% of children with Edwards syndrome [24–26]. Patau syndrome is reported to have cardiac abnormality in 80% of cases with VSD, PDA, ASD and dextroposition [25]. Among 4 patients with Patau syndrome in our study, 3 patients presented isolated VSD, TA and PDA, respectively; while 1 patient had a complexe CHD combining PDA and VSD.

In our series, we found a case of Turner syndrome associated with coarctation of the aorta in which short stature and enlarged webbed neck were predominant. Most of Turner patients have CHD characterized by bicuspid aortic valve in 50% and coarctation of the aorta in 15-30% [27]. The present study detected other well-known genetic syndromes associated with CHD, namely Williams Beuren and DiGeorge syndromes. Williams Beuren syndrome is a rare neurodevelopmental genetic disorder caused in majority of cases by a microdeletion of ELN gene that encodes for elastin. It is clinically characterized by a distinctive, “elfin&#; facial appearance, periorbital fullness of subcutaneous tissues, prominent lips, an unusually cheerful demeanor and ease with strangers; developmental delay coupled with strong language skills, transient hypercalcemia; and cardiovascular problems mainly supravalvular aortic stenosis (SVAS) [28, 29]. The only case of Williams Beuren syndrome identified in our cohort presented a mitral valve prolapse (MVP).

DiGeorge syndrome is a velo-cardio-facial genetic disease caused by a 22q11 deletion. Classically, patients present typical clinical features characterized by hypertelorism, proeminent nose with squared nasal root, thymic aplasia, cleft palate, hypocalcemia, hypoparathyroidism, mental retardation and CHDs predominantly characterized by TOF, interrupted aortic arch (IAA), VSD and persistent truncus arteriosus (PTA) [15, 16]. One patient identified in our series had moderate to severe mental retardation, a long face, forehead, hypertelorism, a proeminent nose with squared nasal root and a large mouth. The echocardiography detected a TOF and the molecular genetic test revealed a deletion of 22q11.21.

In our study, we recruited patients with clinical features of well-recognized syndromes including 4 cases of Noonan syndrome, 1 case of Poland syndrome, 1 case of Frazer syndrome and 1 case of Cornelia Lange syndrome. Unfortunately the molecular analysis could not be performed due to the budget constraints. Among the 4 clinical cases of Noonan syndrome screened for cardiac defect, all had isolated CHD characterized by ASD, AVSD, and PS. This is a high rate of CHDs in these patients compared to other previous studies [11], but this may be due to the few cases of Noonan syndrome in our study. Interestingly, several data showed that PS is the common CHD in patients with Noonan syndrome [30, 31]. We also noticed a case of dextrocardia in a patient with Poland syndrome. In addition, this patient presented unilateral syndactyly and left sided rib defect. Poland syndrome is a rare birth defect characterized by underdevelopment or absence of the chest muscle (pectoralis), rib defect on one side of the body, syndactyly of the hand on the same side (ipsilateral hand), sometimes renal anomaly and dextrocardia.

## Conclusion

In conclusion, our study showed a high incidence of CHDs in Rwandan pediatric patients with genetic defects. VSD, AVSD, PDA, and ASD are the commonest CHDs in our series. Down syndrome is the most commonly identified genetic defect associated with CHD. To our knowledge, this is a first extensive study done in East African population especially in Rwanda demonstrating the pattern and prevalence of CHD associated with genetic defects.
